# The Multidimensional Lexicon of Emojis: A New Tool to Assess the Emotional Content of Emojis

**DOI:** 10.3389/fpsyg.2022.921388

**Published:** 2022-06-10

**Authors:** Rebecca Godard, Susan Holtzman

**Affiliations:** Department of Psychology, University of British Columbia, Kelowna, BC, Canada

**Keywords:** emojis, emoticons, sentiment analysis, computer-mediated communication, online, emotion, lexicon

## Abstract

Emerging studies suggest that emojis can make important contributions to the emotional content and meaning of digital messages. Yet researchers currently lack adequate tools to incorporate emojis into their analyses. To address this gap, we used over 3 million Twitter posts from a period of 17 months and emotion ratings provided by 2,230 human raters to develop and validate the Multidimensional Lexicon of Emojis (MLE). This new lexicon consists of 359 common emojis rated on ten emotion and sentiment dimensions. The MLE is an open-access tool that holds promise for those interested in conducting a comprehensive analysis of the emotional content of digital communication that incorporates emojis and goes beyond the dimensions of negativity and positivity.

## Introduction

Emojis are pictographs representing a wide range of facial expressions, symbols, and objects, and are administered by the Unicode Consortium (Unicode, 2019). Emojis now have a ubiquitous presence in online communication, including text messages, social media, and other online forums, all over the world (Ljubešić and Fišer, 2016; Bai et al., 2019). Emojis play a critical role in emotional communication in online contexts (Bai et al., 2019). In particular, emojis can facilitate communication of subtle emotional cues (e.g., irony, sarcasm, and playfulness; Bai et al., 2019) that may be difficult to convey in more traditional text-based digital communication (Walther, 2011). Accordingly, emojis are often used in communication between people in close relationships (Bai et al., 2019), and they may be an important factor in maintaining emotional closeness and understanding among people who engage in online communication.

Despite the ubiquity of emojis and their known importance to emotional communication, there is currently a lack of measurement tools for researchers to incorporate emojis into analyses of emotion in online contexts. This leads to researchers missing out on critical opportunities to study the emotional functions of emojis, both in specific instances of digital communication and in the broader context of modern social relationships. Therefore, the current study aimed to develop the Multidimensional Lexicon of Emojis (MLE), a new tool to measure the emotional content of commonly used emojis across specific (anger, anticipation, disgust, fear, joy, sadness, surprise, and trust) and non-specific (positivity and negativity) emotional domains.

A number of theories within the field of computer-mediated communication (CMC) have proposed that one of the core limitations of using CMC for interpersonal relationship development and maintenance is that it lacks many of the non-verbal cues available in in-person interactions, such as facial expressions and body language (Culnan and Markus, 1987; Kock, 2004; Walther, 2011). In contrast, social information processing theory (SIP) argues that CMC users can adapt to the cues that are available in order to produce effective interpersonal communications (Walther, 1992, 2011). Emojis are an example of a cue that can help users convey and interpret information that may be otherwise difficult to express using purely text-based communication (Bai et al., 2019). In fact, recent EEG research has found preliminary evidence that emojis displaying facial expressions may be able to elicit similar neural responses as their corresponding “real-life” facial expressions (Gantiva et al., 2019).

A growing body of interview- and survey-based research suggests that emojis are regarded as valuable tools for enriching computer-mediated interactions (Kelly and Watts, 2015). Self-reported motives for using emojis include adding emotional meaning or situational context (Cramer et al., 2016; Holtgraves and Robinson, 2020), communicating indirect meaning (Holtgraves and Robinson, 2020), establishing and adjusting emotional tone (Zhou et al., 2017), reducing ambiguity (Kaye et al., 2016), and communicating friendly intentions (Cramer et al., 2016; Kaye et al., 2016). In an analysis of Spanish WhatsApp users, emojis were found to both upgrade (intensify) and downgrade (soften) speech acts that included emotional content (Sampietro, 2019). They can be used to reduce ambiguity in electronic communication, such as by establishing emotional tone and lightening the mood in a message that could be interpreted as sarcastic or negative (Kaye et al., 2016), or alternatively, by underscoring the presence of irony or sarcasm (Prada et al., 2018). The usefulness of emojis for enriching CMC is not limited to emojis depicting facial expressions, with non-face emojis also proving effective in communicating emotion and disambiguating messages (Riordan, 2017b).

Past research has highlighted other contexts in which emojis may be useful for maintaining and enhancing social relationships *via* CMC. For example, in two lab-based experiments, Riordan (2017a) found that emojis are particularly popular and effective for communicating positive emotions like joy. Similarly, Coyle and Carmichae (2019) recently demonstrated that the use of emojis can lead to more positive impressions and increases in perceived responsiveness when they are used by both conversation partners. Lastly, emojis can make CMC an ideal platform for expressing experiences, emotions, or opinions that are difficult to put into words (Prada et al., 2018). In these instances, perhaps the old adage can be updated to say that an emoji is worth a thousand words.

Taken together, these recent findings have demonstrated that emojis contain information that is necessary for accurately and effectively interpreting the emotional and semantic content of social media posts, text messages, and other forms of CMC. However, to date, the vast majority of research that has examined the emotional content of CMC has failed to incorporate emoji into their analyses. We suspect that the primary reason for this is *not* because researchers dismiss emojis as meaningless images or “noise” in their analyses. Rather, this is likely a reflection of the fact that there has been a dearth of standardized tools available to researchers to be able to evaluate the contribution of emoji in CMC interactions.

Natural language processing (NLP) is an interdisciplinary field that uses computer programs and statistical procedures to extract meaning from human language (Nadkarni et al., 2011). For example, sentiment analysis is a branch of NLP that detects sentiment and/or emotion from language. This has many potential applications, including targeted marketing, tracking attitudes toward an entity, analyzing language to describe group differences or predict behavior, and helping artificial intelligence produce messages with appropriate emotional content (Mohammad and Turney, 2013). One approach to sentiment analysis involves using computer programs to compare the words contained in a text with a lexicon of words known to represent certain sentiments or emotions. These lexicons are typically generated by asking people to rate the extent to which words represent sentiment or emotion categories (Mohammad and Turney, 2013).

Since emojis became widespread, several researchers have devised ways of incorporating them into existing sentiment analysis methodologies. The primary emoji lexicon used for sentiment analysis was developed by Novak et al. (2015), in which human raters classified tweets as positive, negative, or neutral. Sentiment scores for each emoji’s positivity and negativity were calculated as the proportion of tweets containing that emoji rated as positive and negative, respectively. Similarly, Rodrigues et al. (2018) Lisbon Emoji and Emoticon Database (LEED) contains positive–negative valence scores that were derived from human ratings of emojis and emoticons. Another approach to creating emoji lexicons is automatic construction, which uses computer-based techniques rather than human annotators. For example, Fernández-Gavilanes et al. (2018) developed a lexicon by using traditional sentiment analysis to analyze emoji descriptions found on Emojipedia, an online encyclopedia of emojis (Emojipedia, n.d.^1^). Similarly, Kimura and Katsurai (2017) generated an emoji lexicon based on co-occurrence of emojis with emotional words within tweets.

The past 5 years has seen the development of a number of new NLP tools for emotion detection, many of them drawing on deep learning. For example, the DeepMoji model can accurately predict which of 64 popular emojis are appropriate to accompany a short string of text (Felbo et al., 2017). Similarly, the tool emoji2vec produces embeddings, or 300-dimensional vectors used in Google’s word2vec, for all emojis in use in 2016 (Eisner et al., 2016). Although these tools have advantages over a lexicon-based approach, there are several limitations that preclude their widespread application to research in the social and communication sciences. First, their implementation requires a level of technical expertise, particularly familiarity with deep learning methods, that is rare among social and communication scientists. In addition to being inaccessible, deep learning tools are often not suitable for addressing clinically relevant research questions. For example, emoji2vec produces vectors in 300-dimensional space, but these dimensions do not map directly onto theoretical and clinical constructs (e.g., emotions). More typically, research involving emojis requires classifications or scores on defined emotional constructs. Thus, there is a continued and pressing need for the development and validation of emoji lexicons that are both accessible and appropriate for research in social and communication sciences (Bai et al., 2019).

One limitation of existing emoji lexicons is their tendency to focus on a small number of basic sentiments, such as negativity and positivity. Kimura and Katsurai’s (2017) lexicon is an exception, as it includes five emotions (anger, sadness, fear, disgust, and happiness). At the same time, it omits important positive emotions included in other sentiment analysis word lexicons (e.g., anticipation, surprise, and trust). Additionally, their lexicon contains only 236 emojis. In their review of emoji-related research, Bai et al. (2019) call for the development of emoji lexicons that can probe a wide range of complex emotions. This has already occurred within word-based lexicons (e.g., Pennebaker et al., 2015). Of particular relevance to this study, the National Research Council of Canada word-emotion association lexicon (the NRC Emotion Lexicon) developed by Mohammad and Turney (2013) is able to score text on the eight basic human emotions (anger, fear, sadness, disgust, joy, sadness, surprise, anticipation, and trust) as proposed by Plutchik (1960). This lexicon has been used effectively to analyze tweets (Mohammad et al., 2013; Kiritchenko et al., 2014), and is freely accessible through the R package *syuzhet* (Jockers, 2015). While the NRC Emotion Lexicon is useful for sentiment analysis of short texts and provides more information than traditional sentiment analysis, one limitation is its current inability to incorporate emojis. The current study addresses this by developing a lexicon of emojis, designed to be used in conjunction with the existing NRC Emotion Lexicon or other sentiment analysis programs.

## The Current Research

This research developed a lexicon of emojis based on the NRC Emotion Lexicon. It is the first emoji lexicon to include anger, anticipation, disgust, fear, joy, sadness, surprise, and trust, in addition to the basic sentiment categories of negativity and positivity. In Study 1, the MLE was developed based on a sample of over three million English-language tweets from Twitter, a social media platform, which currently has over 300 million active users worldwide (Statista, 2022). These tweets were collected at three time points approximately 17 months apart, and we assessed the reliability of the lexicon scores over time. In Study 2, the convergent validity of the MLE was assessed by examining its consistency with emotion ratings provided by human participants.

### Study 1: Lexicon Development

#### Methodology

The Twitter Stream API allows developers to download a random subset (approximately 1%) of the public tweets produced in the time that the stream is open (Novak et al., 2015). Because these tweets are publicly available, institutional ethics board review was not required. For Study 1, filters were set to include all English-language tweets produced anywhere in the world. We did not collect location data due to the extremely small number of tweets that are tagged with a specific location (approximately 0.85%; Sloan et al., 2013). At the same time, the long duration of data collection (14 days, covering all hours of the day) helped ensure that we obtained a sample of tweets produced by English-speaking (emoji-using) Twitter users from across the world. Retweets and quote tweets were excluded. Using the R packages *streamR* (Barbera, 2018) and *rtweet* (Kearney, 2019), we collected over three million tweets produced at three time points, including 1,014,363 tweets produced between November 7 and November 20, 2019 (Time 1), 1,122,438 tweets produced between September 30, 2020 and November 2, 2020 (Time 2), and 1,021,715 tweets produced between February 20, 2021 and March 4, 2021 (Time 3). Of these tweets, approximately 21% (*n* = 678,879) included at least one of the 1,719 emojis in use in November 2019 (Unicode, 2019). We excluded emojis representing national and regional flags (*n* = 261) due to their highly context-dependent meanings and the potential for their use to be influenced by current events. To ensure that each MLE score was based on an adequate sample of tweets containing that emoji, we retained only emojis that occurred in at least 50 tweets collected at each of the three time points.

Sentiment analysis using the NRC Emotion Lexicon (Mohammad and Turney, 2013) was then run on all unique tweets containing emojis included in the MLE (Jockers, 2015). Each tweet was assigned a score for each of the eight emotions in the NRC Emotion Lexicon – anger, anticipation, disgust, fear, joy, sadness, surprise, and trust. Tweets were also scored on negativity and positivity using the NRC Emotion Lexicon. NRC Emotion Lexicon scores reflect the number of words associated with each emotion in the tweet. For example, a tweet containing four anger-related words and five sadness-related words would receive a score of 4 on anger and 5 on sadness. For each emoji, we classified each tweet in a binary manner as either containing or not containing that emoji. We did not make finer-grain distinctions (e.g., tweets containing one emoji vs. multiple of the same emoji) for two reasons. First, weighting tweets with multiple of the same emoji more heavily would cause the resulting scores to be biased by a small number of tweets containing many of that emoji. Second, research on the use of multiple identical emojis indicates that repetition may not substantially alter meaning (McCulloch and Gawne, 2018), so it remains somewhat unclear how multiple instances of the same emoji should be interpreted in this context. In the next step, each emoji was assigned eight emotion scores and two sentiment scores by averaging the NRC Emotion Lexicon scores of all tweets containing that emoji.

To test the reliability of the lexicon over time, we computed separate scores on the 10 emotion dimensions for each emoji at each of the three time points. We then computed the intraclass correlation (ICC) between emotion scores on each emoji generated at each time point as an index of agreement or stability over time. We computed ICC estimates using the R package *irr* (Gamer et al., 2019) based on a mean-rating (*k* = 3), absolute-agreement, two-way random-effects model (Koo and Li, 2016).

#### Results

##### Included Emojis

A total of 1,458 emoji were identified in the 678,789 emoji-containing tweets. A subset of 359 emojis (25%) met the inclusion criteria of occurring in at least 50 tweets collected at each time point. These 359 emojis made up 87% of the total number of emojis used in the 678,789 emoji-containing tweets in the sample. Smileys and Emotion emojis constituted 56% of the total number of emojis found in the 678,879 emoji-containing tweets and the majority of emojis from the Smileys and Emotion category (*n* = 133; 89%) were represented in our sample of tweets (i.e., they occurred in at least 50 tweets at each time point). In contrast, the other emoji categories had much lower inclusion rates in the lexicon (People and Body: *n* = 59, 19%; Animals and Nature: *n* = 35, 28%; Food and Drink: *n* = 20, 17%; Travel and Places, *n* = 26, 12%; Activities, *n* = 18, 23%; Objects, *n* = 41, 18%; Symbols, *n* = 25, 12%; Flags, *n* = 2; 29%).

##### Sample Validation

To determine whether the emojis found in our sample were representative of the emojis found on Twitter more broadly, we compared the frequency of each emoji in the study sample to the overall frequencies reported by Emoji Tracker in May 2021 (Rothenberg, n.d.). This online tool reports the number of times each emoji has been used on Twitter since July 2013. The Pearson’s correlation between frequency of emoji use in the current sample and frequency reported by Emoji Tracker was 0.91 (*p* < 0.001). The high correlation indicates that the emojis in the sample are representative of the emojis used on Twitter. For example, the “face with tears of joy” emoji was by far the most commonly used in both our sample (*n* = 75,490) and on Emoji Tracker (*n* = 3.27 billion).

##### Reliability Over Time

Across the three time points, ICC values for each emotion were as follows: joy (0.93), positivity (0.87), disgust (0.86), anticipation (0.85), surprise (0.82), anger (0.80), negativity (0.78), trust (0.77), fear (0.77), and sadness (0.71; all *p*’s < 0.001). Based on Koo and Li’s (2016) guidelines for interpreting ICC values, the reliability of the emotion scores over time was excellent for joy and good for all other emotions and sentiments (with the exception of sadness, which was in the moderate range of reliability).

##### Multidimensional Lexicon of Emojis

Given the high ICC values, we averaged the NRC Emotion Lexicon scores of tweets containing each emoji and for each of the 10 dimensions, across all three time points. These scores are what comprise the final MLE. The highest-scoring emojis in terms of level of emotion or sentiment can be found in Table 1. Scores on each of the 10 dimensions, for all 359 emojis in the MLE, can be found in Supplementary Table 1. The lexicon is also currently accessible through the R package *emojis*. Across the 10 measured dimensions, the observed scores for each emoji ranged from 0.05 to 2.80, with higher scores indicating a higher presence of that emotion or sentiment. Table 2 presents examples of tweets that scored high on emotional content or sentiment, with the emotion score generated from the NRC Emotion Lexicon alone, alongside the combined emotion score generated from both the NRC Emotion Lexicon and the MLE. This demonstrates one way in which the new emoji lexicon can be used in conjunction with the NRC Emotion Lexicon (or similar lexicons) to create a score that incorporates the emotional content of emoji(s).

**TABLE 1:** Emojis with highest scores on each emotion/sentiment.

Emotion	Emoji	Description	Score
Negativity	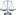	Balance scale	1.03
	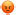	Pouting face	1.01
	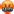	Face with symbols on mouth	0.99
Anger	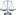	Balance scale	0.68
	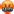	Face with symbols on mouth	0.56
	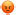	Pouting face	0.55
Disgust	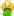	Face vomiting	0.49
	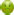	Nauseated face	0.48
	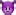	Angry face with horns	0.45
Fear	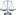	Balance scale	0.68
	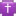	Latin cross	0.63
	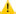	Warning	0.57
Sadness	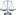	Balance scale	0.55
	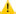	Warning	0.49
	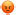	Pouting face	0.48
Positivity	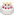	Birthday cake	2.80
	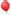	Balloon	2.15
	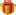	Wrapped gift	2.10
Anticipation	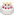	Birthday cake	2.11
	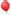	Balloon	1.59
	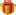	Wrapped gift	1.51
Joy	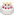	Birthday cake	2.39
	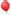	Balloon	1.81
	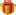	Wrapped gift	1.59
Surprise	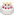	Birthday cake	1.12
	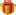	Wrapped gift	0.90
	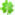	Four leaf clover	0.86
Trust	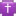	Latin cross	1.55
	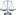	Balance scale	1.39
	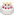	Birthday cake	1.36

**TABLE 2:** Examples of tweets with high emotional content rating using the NRC Emotion Lexicon and the Multidimensional Lexicon of Emojis (MLE).

Emotion	Tweet^a^	Emotion score	
		Words only^b^	Emojis and words^c^
Negative	Dirty, filthy stinking vermin. The human race disgusts me. Cruel and barbaric. The amount of evil that walks amongst us is staggering. 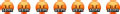	8	14.94
	By my count I’m on day 9 of a fibro flare and there are minimal signs of it getting any better 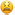 It is now also impossible to eat most things with a shattered tooth (that I have to wait 2 weeks to get fixed 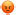 ) and a gap where my old incompetent dentist ripped one out…	5	6.53
Anger	People hating on [mention] ye are sick bunch, support him or don’t say [expletive]! Mans looking for this fight for years even changed promotion to get the canelo fight. Don’t hate appreciate!! Best of luck champ and massive respect to you brother. Bring on May! 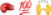	4	5.00
	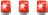 BREAKING: Fraud Scheme Steal Mich 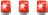 Detroit training in October: “corruption, crime from STATE EMPLOYEES.” “lie to voters, destroy ballots, disenfranchise poll challengers, & call cops to whom object…” 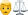 [mention] Add @ Follow ALL & [mention] Bkmark∼Rtrn	4	7.30
Disgust	[mention] Well, I hate to burst your bubble but a lot of capitalists are VOTING FOR [mention]….and so is most of America. WE ARE ALL UNITED TO DEFEAT A PIECE OF GARBAGE TRAITOR MURDERER WOMAN ABUSER frump. So go peddle your “socialist” BS somewhere else! ??????#VoteBidenHarrisToSaveAmerica	5	5.10
	That’s just out 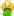 right nasty 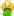 & 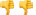 disgusting!!!To treat a 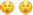 PERSON WITH SUCH HORROR 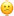 …Sooo Sickening 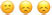 & Sad!!!	5	8.19
Fear	THESE ANTIFA THUGS DON’T REALIZE THAT THEY MIGHT BE ATTACKING SOMEONE WITH A CONCEAL-CARRY GUN PERMIT, THEN WHAT IF THE GUY BEING ATTACKED STARTS SHOOTING. ONE DAY AN ANTIFA THUG IS GONNA GET SHOT. THEY BETTER STOP HARASSING PEOPLE B4 SOMEONE GETS SHOT 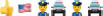	5	5.22
	Hey friends…tomorrow Mo is having surgery to remove a tumor (hopefully not cancerous, but the vet just wants to be sure) so if you could send some good vibes our way…I know he’ll be fine but I’m still pretty nervous ??????	4	4.55
Sadness	Several Republican candidates in Central Florida are going negative, attacking their Democratic women opponents with mail, TV ads, and in my case a trash website. 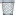 You know you’re winning when they go negative. And with every act of intimidation our courage only rises. 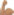	5	5.20
	Yes this is America. The America many know all our lives. Keep on as best can. Maybe with hope, as some who don’t know as we do look to see what we see. They work to change it. Now everyone can see. More work to stop inequities hate violence. Sadly, more are targeted too 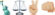	3	4.04
Positive	Happy 30th Birthday Hope you have an amazing day, full of love, fun, joy and happiness! It’s what you deserve 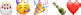	9	17.77
	Happy birthday to my [mention] 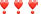 Wishing you all the best in studies, health and wealth 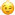 I love how you keep asking me questions so please continue to do so. Have a blast and stay safe 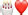	6	14.77
Anticipation	Birthday boy reached his favorite destination to celebrate birthday 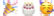 See ya guyz tomorrow same time to celebrate the most fav day of our year 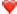 hope to see most of you 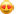 for details check fleet. Can’t wait for the fun celebration 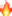	8	14.15
	#BIRTHDAYPRINCESS Happy Happy 2nd Birthday To My Smart & Super Pretty Daughter Avery Daniella Francisco, The Most Precious Gift God Has Given To Me. I Wish U Have More Birthdays To Come. All I Want Is To Give All Of U The Life That You Deserve. I Love You Very Much 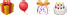	7	13.56
Joy	Happy Happy Birthday 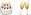 I pray that you always find happiness in all the things that you do and have. Hope you are spending your day w/the people you love. Enjoy your day, eat lots of good food. U are loved, thank u for being born 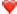 #HappyLeoDay	9	13.25
	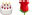 Dear Ann, Wishing you a Happy Birthday. Many joyful times to you. You cake looks so very beautiful. Know you were surprised. Enjoy your special day. Many, many more. Happy Birthday. XOXO. God bless you. 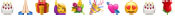	8	24.82
Surprise	#BognorMindShop has a #Lovelinks necklace, valued at £70! You have a chance to WIN IT IN A #RAFFLE – ONLY £2 PER TICKET! This could be the perfect #Christmas #gift, for someone special in your life… 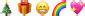	3	5.37
	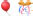 Congratulations to our Mullica Hill Community & Real Estate News Fall Coloring Contest Winner! 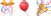 She won a basket full of art supplies and a gift card to Cherry on Top! 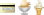 If your child entered, keep…	3	6.74
Trust	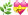 Good morning, Dear Sister. Thank you. Hope you are doing well. Happy, blessed Thursday. Enjoy your morning coffee. Can relax a little more because of being home, hopefully. Night. Take care. See you soon. Love you. God bless you and keep you safe. 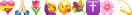	7	16.93
	This comment will stick with me forever. Thank you for being such a good and supportive friend, and just being you. Hope you have the best birthday 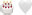	3	5.09

*^a^Weblinks, mentions, and profanities have been removed.*

*^b^Emotion scores generated from an analysis of the tweet using the NRC Emotion Lexicon (words only).*

*^c^Emotion score generated by adding the score from the MLE (emojis only) to the score from the NRC Emotion lexicon (words only).*

The 359 emojis in our lexicon are presented in descending order of frequency in Supplementary Table 1. For the sake of brevity, we list the top 10 most frequently used emojis here, starting from the most popular: face with tears of joy, loudly crying face, red heart, rolling on the floor laughing, pleading face, folded hands, smiling face with heart-eyes, fire, smiling face with hearts, and sparkles. The top 10 emojis scored significantly higher on positive sentiment than negative sentiment, *t*(10.7) = 5.81, *p* < 0.001, *d* = 2.60. More specifically, scores on these emojis tended to be higher for positivity (0.61–1.47), anticipation (0.33–0.78), joy (0.30–0.88), and trust (0.38–0.98), and lower on anger, disgust, fear, sadness, and surprise (with scores consistently below 0.36).

### Study 2: Validation Using Human Raters

In Study 2, we conducted an initial validation of the MLE by evaluating the similarity of MLE scores (derived using the NRC lexicon) with emotion scores generated by human raters.

#### Method

Raters were recruited from an undergraduate subject pool at a university in Western Canada. Eligible participants were at least 18 years old, spoke English fluently, and used emojis in digital communication (e.g., texting and social media) at least once per week. Participants received course credit for completing the study. Data was collected between November 2020 and November 2021.

Study participants completed an online survey *via* Qualtrics. Participants were asked to rate a series of emojis on the eight emotions included in the MLE (anger, anticipation, disgust, fear, joy, sadness, surprise, and trust). Specifically, they were asked, “to what extent does this emoji communicate the following emotions?” using a 4-point Likert scale, ranging from 1 (not at all) to 4 (a lot). They were also asked to rate each emoji’s positivity and negativity on the same 4-point scale.

In this initial validation, we collected human ratings for the 133 emojis in the Smileys and Emotions category. To reduce fatigue, each participant provided ratings on a random subset (*n* = 50) of the 133 Smiley and Emotion emojis. Consequently, each of the 133 emojis was rated by between 335 and 665 participants. Scores for each emoji on each emotion or sentiment were generated by averaging the ratings provided by each participant.

The Smileys and Emotions category of emojis includes faces with various expressions, hearts, and other emotion-related symbols (i.e., stars and bomb). We did not collect human ratings on the emotional content of emojis from other categories, including People and Body (e.g., “backhand index pointing up”), Animals and Nature (e.g., “palm tree”), Food and Drink (e.g., “pizza”), and Activities (e.g., “baseball”). On a practical level, we anticipated that participants would have a difficult time producing reliable and useful emotion ratings for many of these emojis provided in isolation (e.g., the degree of sadness represented by a single piece of fruit or baseball), and they were not developed with this purpose in mind. Indeed, recent research has shown that face emojis are more frequently used to represent abstract concepts, compared to concrete concepts (Wicke and Bolognesi, 2020).

#### Results

A total of 2,230 participants (75% female, 24% male, 1% non-binary or other; age: *M* = 20.3, SD = 2.9) completed the online survey. Almost all participants (99.9%) owned smartphones (87% Apple, 12% Android, <1% other), and the majority reported using emojis frequently in text messaging (*M* = 3.9, SD = 0.9), social media messaging (*M* = 4.1, SD = 1.1), and social media (*M* = 3.5, SD = 1.3; all 5-point Likert scales, 1 = never, 5 = very frequently). Emotion ratings provided by participants displayed moderate positive skew, yet there were minimal outliers. The average proportion of outliers (>3 SD from the mean) across emojis and emotions/sentiments was 1.5%. Running analyses with and without these outliers did not produce significantly different results. Therefore, we present findings based on the full data set containing a small number of outliers.

Due to the moderate positive skew of the human-generated scores, Spearman’s correlations were used to determine the strength of the association between the human-generated and automatically generated scores on the eight emotions and two sentiments. The correlation coefficients were as follows: negativity (0.83), disgust (0.83), sadness (0.75), anger (0.73), fear (0.73), joy (0.71), positivity (0.61), trust (0.51), surprise (0.28), and anticipation (0.20; all *p* < 0.05). According to Carlson and Herdman’s (2012) guidelines, these values can be considered good for negativity, disgust, sadness, anger, fear, and joy, adequate for positivity and trust, and inadequate for surprise and anticipation.

## Discussion

The current research presents the development and initial psychometric evaluation of the MLE. For 359 of the most commonly used emojis, this new lexicon produces scores on eight emotions (anger, anticipation, disgust, fear, joy, sadness, surprise, and trust), as well as positive and negative sentiment. This information can be used independently, or easily incorporated into any sentiment analysis of digital communication containing emojis, including text messages, emails, and social media posts. Approximately one-quarter of the 3,158,516 tweets collected in this study contained at least one emoji, reflecting the widespread popularity of their use in digital communication. This emoji lexicon holds great promise for those interested in conducting a comprehensive, nuanced analysis of the emotional content of digital communication that (1) incorporates emojis and (2) goes beyond the dimensions of negativity and positivity.

Overall, the 359 emojis included the MLE displayed strong consistency in their emotional connotations across three separate time points, over a 17-month period. This is noteworthy given previous suggestions that emojis may take on new meanings over time and across demographic groups (Robertson et al., 2021). The stability of the MLE scores was particularly impressive for positive emotions such as joy (ICC = 0.93), and positive sentiment overall (ICC = 0.87). This suggests that emojis with positive emotional connotations tend to be used very similarly over time. The stability for negative sentiment (ICC = 0.78) and negative emotions (e.g., disgust, ICC = 0.86; anger, ICC = 0.80) was also good. Sadness was the only dimension of the MLE that performed in the moderate range of stability (ICC = 0.71). While further research is needed, these differences in stability may be linked to world events, particularly COVID-19 pandemic, that unfolded between Time 1 (November 2019) and Time 2 (September–November 2020). In this context, certain negatively valenced emojis (e.g., “face with medical mask” and “syringe”) may have accrued new or stronger emotional connotations. It is also possible that emojis are less frequently used to express sadness in digital communication (Prada et al., 2018). There may also be greater intra- and interpersonal variability in terms of which emojis are chosen to express sadness, particularly compared to emotions such as joy. In other words, people may consistently choose the same emojis (e.g., “balloon” and “birthday cake”) to express joy, but may use different emojis to express sadness based on contextual and individual (e.g., personality and experience using emojis) factors.

Findings from Study 2 also revealed a high level of agreement between the emotion ratings of emojis generated from Study 1 (using NRC analysis of Twitter posts) and those obtained from a sample of over 2000 human raters (who were asked to provide emotion ratings for a list of individual emojis). Agreement was particularly high for joy, negative emotions (anger, fear, disgust, and sadness), and negative sentiment (all correlations were 0.70 or higher). On the other hand, agreement between human raters and scores on the MLE was quite low for surprise and anticipation (ρ = 0.28 and 0.20, respectively). Lower scores for anticipation may relate to the greater complexity of this emotional state, and perhaps a lower tendency for people to use emojis to convey anticipation in digital communication. The low agreement found for surprise is more puzzling, particularly given the number of face emojis that appear to explicitly communicate surprise (e.g., “astonished face,” “face with open mouth,” and “face with crossed-out eyes”). Given the findings of Study 2, researchers are urged to use caution when using the MLE to estimate levels of surprise and anticipation in their data. It is also important to note that discrepancies in the ratings between Study 1 and Study 2 may have been related to the smaller, homogeneous sample of human raters (university students in Western Canada) in Study 2, compared to the Twitter users that composed the tweets in Study 1. Further, since emojis are often used in conjunction with text-based communication, human raters may also have had difficulty rating the emotional content of emojis when presented in isolation.

The current research demonstrates that researchers are likely missing out on important emotional information by failing to include emojis in their analyses. For example, in Table 2, both disgust tweets received a disgust score of 5 when analyzed without emojis, but they received scores of 5.10 and 8.19 when the MLE was used to incorporate the emotional content of emojis. That is, both tweets would have received the same score using word-only sentiment analysis, but when emojis are taken into account, we see a significant divergence in the emotional intensity of these messages. Researchers have started to use Twitter and Facebook posts to predict important outcomes and events, including depression and suicide (De Choudhury et al., 2013; Won et al., 2013). By taking into account the emotional content and sentiment of both text and emojis, we can likely improve the predictive power of these models. Since this new lexicon generates continuous numerical scores for each emoji on 10 different dimensions, it also offers greater precision than a binary classification that identifies whether or not a word represents a specific emotion or sentiment, which has been used previously (Mohammad and Turney, 2013). Nonetheless, continuous scores from our lexicon could certainly be converted to a categorical system by selecting a cut-off point, if that better suits the goals of the research.

Additionally, incorporating emojis into sentiment analysis can help clarify texts with ambiguous emotional contents. One of the primary functions of emojis in CMC is reducing ambiguity, clarifying indirect meaning, and conveying complex emotions like sarcasm (Kaye et al., 2016; Holtgraves and Robinson, 2020). Particularly in short texts like social media posts or text messages, it may be difficult for researchers to detect emotional content using traditional linguistic analysis techniques. While sophisticated tools for analyzing short texts exist, harnessing the emotional content of emojis using the MLE may provide a simple and accessible approach to disambiguating short texts. For example, the message “You deserve it” could be intended positively (e.g., congratulating a friend on an accomplishment) or negatively (e.g., enjoying another’s misfortune). Taking emojis into account can help determine the emotional content of this ambiguous text. For example, if it is followed by a smiley face and a balloon, it was likely intended positively. However, if it is followed by an angry face and a balance scale, it was likely intended negatively. Thus, emojis are an important component of understanding the emotional content of social media posts, text messages, and other texts containing emojis.

Findings from the current research also make important contributions to the broader study of emojis and emotions in online communication. In our sample of 678,879 tweets containing emojis, the majority of emojis (58%) came from the Smileys and Emotion category. Furthermore, nearly three-quarters of the tweets containing emojis included at least one emoji from this category. In addition to finding that emojis in the Smileys and Emotion category were highly prevalent, our study found that the most commonly used emojis were overwhelmingly positive in emotional content, displaying high scores on positivity, anticipation, joy, surprise, and trust. This parallels earlier findings that emojis are particularly important for the communication of positive emotions (Riordan, 2017a). Along these lines, Prada et al. (2018) found that people more often use emojis to make a message more positive, fun, and comical, than they do to make a message more negative or serious. Thus, our finding that people most commonly use emojis in a way that conveys positivity, anticipation, joy, and trust supports the broader hypothesis that emojis are particularly popular for communicating positive emotions in a computer-mediated context.

It is also worth noting that there are several emojis in our lexicon that received very high scores on every positive dimension. For example, the emojis “birthday cake,” “wrapped gift,” and “balloon” were the three highest-scoring emojis for positivity, anticipation, joy, and surprise. While this may initially raise concerns about specificity, we see these emojis’ high scores on multiple positive emotions as indicating their utility for expressing a variety of positive emotional states and perhaps that they are used most consistently in positive contexts and situations. Furthermore, we did see divergence on specific positive emotions when looking at emojis with high (but not the highest) positive emotion scores (e.g., “bouquet” scores high on joy, “fireworks” on anticipation, “four leaf clover” on surprise, and “graduation cap” on positivity). This indicates the MLE’s ability to distinguish between emojis with indiscriminately positive connotations (e.g., “birthday cake,” “wrapped gift,” and “balloon”) and those with more specific meanings (e.g., “bouquet,” “fireworks,” “four leaf clover,” and “graduation cap”).

### Limitations and Future Directions

While this study provides a valuable step toward developing a large-scale, broadly applicable tool for analyzing the emotional content of emojis, it has several limitations. First, the use of a large, publicly available sample of digital communication on Twitter meant that we could not access tweeter’s actual experienced emotions at the time of their tweet, or the emotions they intended to communicate through their tweet. Future research is needed to validate the MLE against the emotional experience of the senders themselves, and how emojis can influence the emotional tone of digital messages from the perspective of the recipient. Related to this, we also assumed that emojis contained in a tweet matched the emotional content of the words of the tweet. Although this is likely to be generally true, there is some evidence that emojis can be used strategically to shift the tone or emotional content of a message (e.g., by indicating sarcasm; Kelly and Watts, 2015; Cramer et al., 2016; Kaye et al., 2016; Prada et al., 2018). While this does reflect a limitation of our research, our approach also had strengths. The use of automatically generated emotion ratings enabled us to evaluate 678,879 tweets containing emojis, which is far beyond what would be possible using human raters, and served to enhance the reliability of the MLE scores. In casting a large and wide net, we were also able to capture the diversity of ways in which emojis are naturally used among English-speaking Twitter users all over the world, thus enhancing the external validity of our results. The relatively high levels of agreement between MLE scores and human ratings derived in Study 2 further support the utility of this approach. However, further research is needed to better understand how emojis are used to shift emotional tone or content (not just complement it), and to consider the potential of integrating both human and automated ratings into the MLE.

Similar to much past research on emoji use (Bai et al., 2019), we collected data from Twitter to develop our lexicon of emojis. This stands as another limitation of this research as it may not paint a representative picture of overall emoji use. While Twitter users are representative of the general population on some demographic variables (e.g., gender), they are also more likely to be young and middle-aged adults, educated, and higher income (Pew Research Center, 2021). At the same time, these demographic patterns are also observed for other social media platforms, indicating that our data may be more representative of emoji users compared to the general population. Some research also indicates that Twitter users use fewer positive emojis than Facebook users (Tauch and Kanjo, 2016), others find negligible between-platform differences in emoji use (Kaye et al., 2016). Additionally, we are not aware of any research that addresses between-platform differences in the emotional content of specific emojis. Thus, while this lexicon may be optimized for use with Twitter data and populations similar to Twitter users, we believe that it is also useful for analyzing texts from other sources (i.e., other social media platforms, text messaging, etc.). Additionally, while the lexicon contains all emojis with a 0.005% or higher incidence rate in the current sample, it may miss out on emojis that are emotionally significant despite their infrequent use. In particular, emojis depicting national and regional flags may be used in highly emotional contexts, yet their content is difficult to quantify due to their frequent use in the context of current events, holidays, and other time-sensitive circumstances. Future research could also investigate culture-dependent use of emojis, as past research has identified cultural differences in emoji use (Guntuku et al., 2019).

A challenge in emoji research is that the meaning of emojis shifts and evolves over time and can be impacted by factors like political climate and social trends. While this raises a possible concern about the stability of the MLE in the future, the large-scale, worldwide data collection used in developing this lexicon likely minimized the influence of idiosyncratic events on its overall reliability and validity. The high degree of correlation between scores generated at three time points over a span of 17 months further supports the long-term usefulness of the MLE. Nonetheless, we plan to update this lexicon annually to help account for trends in emoji associations and meanings, as well as new emojis that have been released by Unicode.

Our study was also limited by only including emojis from the Smileys and Emotion category in the human raters analysis of Study 2. We made this choice due to the potential for emoji users to have difficulty identifying the emotional content of non-face emojis when viewed out of context, despite their importance in emotional communication (Riordan, 2017b), use in representing abstract and concrete concepts (Wicke and Bolognesi, 2020), and inclusion in other emoji lexicons (Novak et al., 2015). At the same time, future research could use more complex methods to obtain human ratings for the emotional content of non-face emojis, such as asking raters to match emojis to posts or messages with known emotional valences. Additionally, future research could investigate the use of emojis among people from a broader range of emoji users. While the tweets in our sample were likely representative of English-language tweets posted on Twitter during the time of data collection, we were not able to reliably extract demographic information such as location, culture, gender, or age. Some past research has uncovered age (Hsiao and Hsieh, 2014; Gallud et al., 2018) and gender (Jones et al., 2020) differences in emoji use. Therefore, future research is needed to investigate differences in the emotional content of emojis produced by people in different demographic categories.

Future research could also investigate the effects of using multiple emojis within the same tweet or message, including using combinations of emojis (e.g., an angry face followed by a laughing face) or multiple of the same emoji (e.g., five disgusted faces in a row). Unlike words, emojis are often repeated for emphasis. For example, McCulloch and Gawne (2018) found that approximately half of the most common sequences of two, three, and four emojis produced on smartphone keyboards consist of pure repetition of one emoji. Repeated emoji use may affect the emotional intensity of a message in a way that is not directly correlated with the number of emojis, and this may be affected by individual and contextual differences such as personality, age, and platform. Thus, further research is needed to better capture the emotional implications of emojis used in conjunction with each other, not just in conjunction with words.

## Conclusion

In summary, emojis are an important component of nonverbal, emotional communication within CMC. Historically, sentiment analysis has not been able to take emojis into account when analyzing social media posts, text messages, and other texts containing emojis, thereby missing out on key indicators of emotion. The current research has produced an emoji lexicon that can be used independently, or in conjunction with an existing linguistic or sentiment analysis tool. Based on a sample of 678,789 emoji-containing tweets collected across a 17-month period, the MLE provides numerical ratings of the 359 most common emojis (all present in >0.005% of the tweets in the sample) on eight emotions and two sentiment dimensions. Despite a rapidly evolving landscape of digital communication, the impressive stability of the MLE over time indicates the viability of this lexicon to retain relevance and utility in the future.

## Data Availability Statement

The original contributions presented in this study are included in the article/Supplementary Material, further inquiries can be directed to the corresponding author.

## Ethics Statement

The studies involving human participants were reviewed and approved by the University of British Columbia – Okanagan Behavioural Research Ethics Board. The participants provided their written informed consent to participate in this study.

## Author Contributions

RG was responsible for study conceptualization and design, data collection and analysis, initial manuscript writing, and manuscript revision. SH was responsible for supervision, study design, and manuscript revision. Both authors contributed to the article and approved the submitted version.

## Conflict of Interest

The authors declare that the research was conducted in the absence of any commercial or financial relationships that could be construed as a potential conflict of interest.

## Publisher’s Note

All claims expressed in this article are solely those of the authors and do not necessarily represent those of their affiliated organizations, or those of the publisher, the editors and the reviewers. Any product that may be evaluated in this article, or claim that may be made by its manufacturer, is not guaranteed or endorsed by the publisher.

## Footnotes

1
https://emojipedia.org
